# Graphene based hyperbolic metamaterial for tunable mid-infrared biosensing

**DOI:** 10.1039/d0ra09781k

**Published:** 2021-02-17

**Authors:** Sarah Cynthia, Rajib Ahmed, Sharnali Islam, Khaleda Ali, Mainul Hossain

**Affiliations:** Department of Electrical and Electronic Engineering, University of Dhaka Dhaka-1000 Bangladesh mainul.eee@du.ac.bd; School of Medicine, Stanford University Palo Alto California 94304 USA

## Abstract

Plasmonic biosensors, operating in the mid-infrared (mid-IR) region, are well-suited for highly specific and label-free optical biosensing. The principle of operation is based on detecting the shift in resonance wavelength caused by the interaction of biomolecules with the surrounding medium. However, metallic plasmonic biosensors suffer from poor signal transduction and high optical losses in the mid-IR range, leading to low sensitivity. Here, we introduce a hyperbolic metamaterial (HMM) biosensor, that exploits the strong, tunable, mid-IR localization of graphene plasmons, for detecting nanometric biomolecules with high sensitivity. The HMM stack consists of alternating graphene/Al_2_O_3_ multilayers, on top of a gold grating structure with rounded corners, to produce plasmonic hotspots and enhance sensing performance. Sensitivity and figure-of-merit (FOM) can be systematically tuned, by varying the structural parameters of the HMM stack and the doping levels (Fermi energy) in graphene. Finite-difference time-domain (FDTD) analysis demonstrates that the proposed biosensor can achieve sensitivities as high as 4052 nm RIU^−1^ (refractive index unit) with a FOM of 11.44 RIU^−1^. We anticipate that the reported graphene/Al_2_O_3_ HMM device will find potential application as a mid-IR, highly sensitive plasmonic biosensor, for tunable and label-free detection.

## Abstract 2

Graphene/Al_2_O_3_ HMM stack, coupled to a gold grating structure, for tunable, label-free, and highly sensitive biosensing in the mid-IR wavelength region. FDTD simulations show that the proposed device can achieve a maximum sensitivity of 4052 nm RIU^−1^.

## Graphical abstract

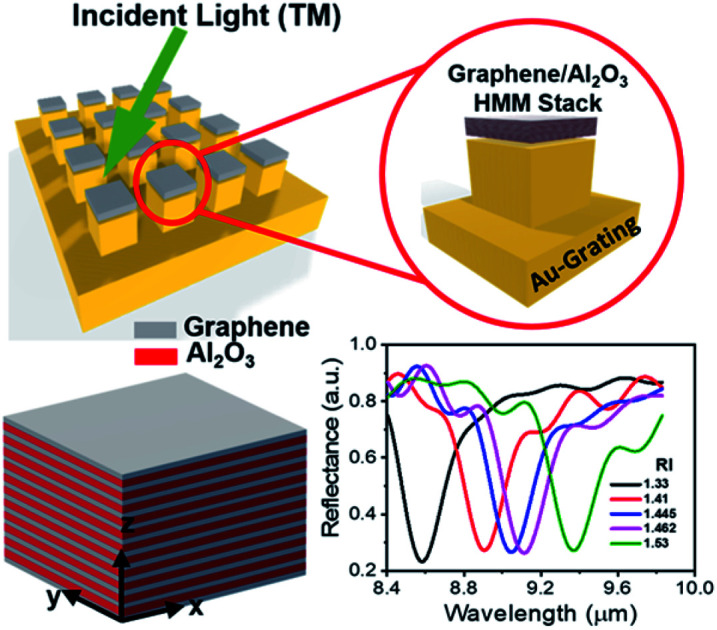

## 1. Introduction

Plasmonic biosensors have been extensively used for rapid, real-time, and label-free detection of biomolecules, at ultralow concentrations.^1,2^ These devices, primarily targeted for point-of-care (POC) applications, rely on the excitation of coherent oscillations of delocalized conduction band electrons, when light, incident on a metal/dielectric interface, meets the desired resonance conditions.^3^ In macroscopic thin metal films, the resulting charge density oscillations, known as surface plasmon polaritons (SPP), propagate along the boundary of the metal and the dielectric and decay exponentially in the transverse direction.^4^ For nanostructured surfaces, the electric field is confined in the vicinity of the nanostructure, giving rise to localized surface plasmon resonance (LSPR). At visible and mid-infrared (mid-IR) wavelengths, the electric field, associated with the surface plasmons, is highly sensitive to change in refractive index (RI) of the surrounding medium. The interface RI change may be caused by the adsorption of biomolecules onto the sensing platform, leading to a shift in the resonant wavelength. The sensitivity of plasmonic biosensors is defined as the resonance wavelength shift per refractive index unit (RIU) and can be increased by enhancing the electric field on the metal surface. Sensor performance, in the detection limit, is evaluated in terms of the figure of merit (FOM) which is expressed as the ratio of sensitivity to the full width at half-maximum (FWHM) of the resonance dip.^5^

Conventional, state-of-the-art plasmonic biosensors make use of metallic nanostructures to confine the electric field within 5–15 nm of the nanostructure surface, giving rise to near-field enhancements through LSPR.^6^ This enables LSPR based plasmonic biosensors to detect small-sized molecules with high sensitivity.^7^ However, due to high carrier concentrations, metal based plasmonic sensors are more responsive in the visible and ultraviolet (UV) wavelengths.^3^ On the other hand, absorption in the mid-IR wavelength region (∼3–20 μm) offers the unique capability to probe molecular vibrations that are characteristic of the bonds in the biological samples.^8,9^ Therefore, plasmonic sensors operating in the mid-IR spectral region, can offer high specificities by detecting the vibrational fingerprints associated with the molecular bonds.^10,11^ Although, metal based plasmonic biosensors have gained considerable success due to simple, miniaturized, low-cost optical setup, and label-free sensing capabilities, their use is limited by high optical losses in the metal, followed by reduced spectral bandwidth and weak electric field confinements in the mid-IR ranges.^12^ Moreover, overcoming the large mismatch, between the IR wavelengths and the nanometre size of the biomolecules, presents a considerable challenge, particularly for detecting samples with ultralow concentrations.^13–15^ In addition, traditional, metal-based plasmonic sensors suffer from low detection speed and limited external tunability.

Recently, graphene is being explored as a promising candidate for plasmonic biosensors.^16–24^ The two-dimensional (2D) nature of graphene has been shown to support stronger, deep-subwavelength confinement of plasmons in mid- and far-IR part of the spectrum, where the plasmons can be dynamically tuned through electrostatic gating, chemical doping, or modulation of graphene structure. In addition to broadband tunability, graphene plasmons are associated with longer lifetimes and lower losses.^25^ Furthermore, high surface-to-volume ratio of graphene and π-stacking interactions, between the 2D hexagonal graphene cells and the carbon-based ring structure of the biomolecules, strongly adsorb the biomolecules onto graphene surface, aiding the biosensing process.^26–28^ Several forms of graphene based plasmonic biosensors have been proposed, including hybrid metal–graphene structures. The seminal work on mid-IR graphene biosensors, by Rodrigo *et al.* used graphene nanoribbons to sense vibrational fingerprints of protein molecules, where the plasmonic resonance was electrostatically tuned.^17^ Based on plasmon-induced transparency phenomenon, Vafapour *et al.* designed a broadband, mid-IR, graphene biosensor, with three slot antennas, achieving an optical sensing coefficient of 99%.^19^ Besides, Hong *et al.* combined asymmetric gold (Au) nano-antennas and unpatterned graphene sheets to achieve multi-functional, broadband sensing, covering both near-IR and mid-IR wavelengths.^16^ Optical conductivity based mid-IR sensors, with ultrahigh sensitivity, was reported by Zhu *et al.* through the use of Au nanorod antenna array covered by monolayer graphene.^29^ Furthermore, the study by Wu *et al.* employed a graphene sheet, integrated on top of a Au grating structure, for mid-IR sensing of vibrational modes from protein molecules.^20^

Plasmonic biosensor platforms, using hyperbolic metamaterials (HMMs), have gained considerable attention in recent times for their extreme broadband sensitivity, arising from enhanced light–matter interactions.^30–32^ HMMs are artificially engineered, strongly anisotropic metamaterials, which exhibit hyperbolic dispersion with one of the principal components, either the permittivity or the permeability, having a negative sign. The hyperbolic frequency dispersion in HMMs allows the propagation of highly confined wavevector modes (high-k modes), across a metal/dielectric multilayer structure. These high-k modes are known as bulk plasmon polaritons (BPPs) and decay exponentially outside the structure. BPPs lead to unusual properties of the HMMs, with extraordinary applications like negative refraction, subwavelength imaging, spontaneous emission enhancement, nanoscale light confinement and biosensing.^33–35^ In practice, highly sensitive HMM biosensors have been realized using alternating layers of metal and dielectric. Each metal/dielectric bilayer evanescently couples the short range, propagating SPPs to its adjacent bilayer, leading to the existence of BPPs. Replacing the metal with graphene, can lead to new possibilities in terms of stronger plasmon response in mid-IR range, smaller material loss and tunable conductivity.^36–38^ Furthermore, owing to the atomically thin nature of graphene, HMM sensors based on graphene can offer extreme scalability, which is important in realizing portable, POC biosensors. Although, graphene based HMMs have been used in a wide variety of applications, such as, exhibition of negative refraction,^39^ switchable reflection modulator,^40^ and perfect absorbers,^41^ their use, in biosensing platforms have been limited.

In this work, we propose a novel HMM biosensor, consisting of a multi-stack of graphene/Al_2_O_3_ bilayers, patterned on top of a gold grating structure with rounded corners, to introduce plasmonic hotspots and enhance sensitivity. The HMM stack and the sub-wavelength nanograting structure effectively couples incident light into the high-k modes in graphene by mitigating the large *k*-vector mismatch between propagating modes in free space and in the HMM slab. Moreover, the resonance wavelength can be tuned by changing structural parameters of the HMM stack and the grating, as well as the Fermi energy of graphene. For the biosensor, proposed in this study, resonance wavelength shift, caused by refractive index (RI) change, is calculated, using finite-difference time-domain (FDTD) simulations, in three-dimensions (3D). All simulations were carried out using the commercially available Lumerical software package. The sensor performance is numerically evaluated, in terms of both sensitivity and FOM, for a wide range of RIs that correspond to commonly used biomolecules.

## 2. Modelling and methods

**Fig. 1:**
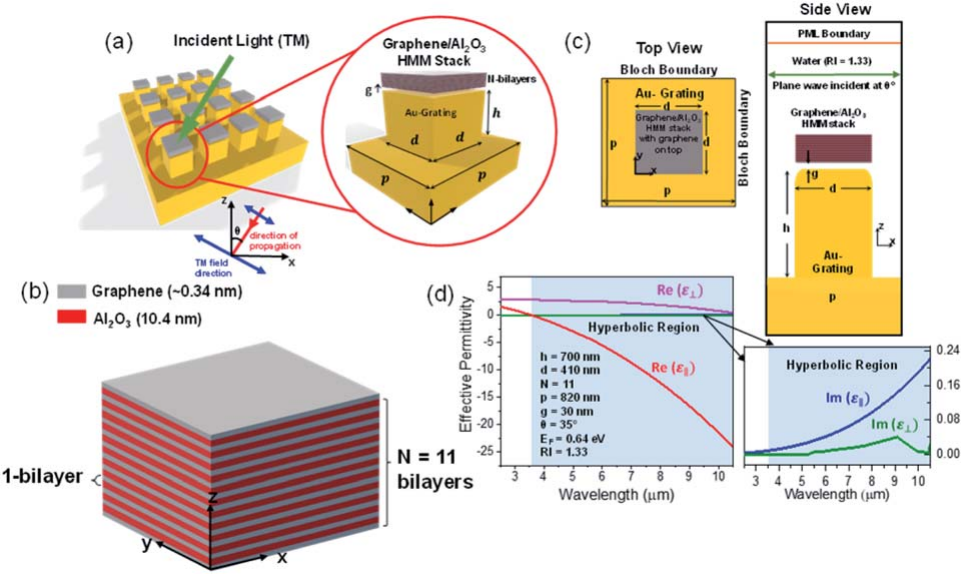
(a) Schematic representation of the proposed HMM biosensor stack on top of a gold grating structure (b) graphene/Al_2_O_3_ HMM stack with *N* = 11 bilayers and a graphene layer on top (c) top and side view of a unit cell used in the simulation (d) real and imaginary parts of effective permittivity of graphene/Al_2_O_3_ HMM, with 11 bilayers, determined with effective medium theory. Hyperbolic dispersion occurs when *λ* > 3.58 μm. Here, *p*, *g*, *d*, and *h* denote the period, radius of the rounded corner, width, and height of the grating structure, respectively.

Fig. 1(a) shows the 3D schematic of the proposed HMM device, consisting of *N* bilayers of graphene/Al_2_O_3_, placed on top of the Au grating structure, with rounded corners of radius *g*. Several studies in the past have fabricated and characterized grating structures, with rounded corners, for a variety of applications.^42,43^ In the proposed design, the grating height, *h*, is fixed at 700 nm while the width, *d*, changes with change in grating period, *p*. For the structure in Fig. 1, *p* = 820 nm, *d* = 410 nm. The topmost layer of the HMM stack, which forms the sensing surface, consists of a graphene sheet, as shown in Fig. 1(b). Using graphene as the sensing surface greatly enhances the sensitivity of the proposed sensor. Top and side views of the unit cell, simulated with periodic (Bloch) boundary conditions, are shown in Fig. 1(c). 2D monolayer of graphene sheets with thickness, *t*_g_ ∼ 0.34 nm, are separated by Al_2_O_3_ layers of thickness, *t*_d_ = 10.4 nm, satisfying the metamaterial limit.^42^ The number of graphene/Al_2_O_3_ bilayers, *N*, can be varied to optimize the absorption properties. Absorption by the HMM structure, can also be tuned, by changing *p* and *g* of the grating structure, and the doping levels (Femi energy, *E*_F_) in graphene. To avoid diffraction in free space and excite high-k modes in the graphene HMM slab, *p* is always kept within the sub-wavelength dimension. The incident light, in the mid-IR range, is transverse magnetic (TM) polarized and irradiates the device from the top at an angle, *θ*. TM mode is chosen because, in general, it shows higher sensitivity than the transverse electric (TE) mode.^32^

The proposed graphene/Al_2_O_3_ HMM structure in the FDTD simulation was described *via* dielectric functions of Al_2_O_3_ and surface conductivity of graphene. The uniaxial dielectric tensor components of this anisotropic HMM can be approximated as:^40^1
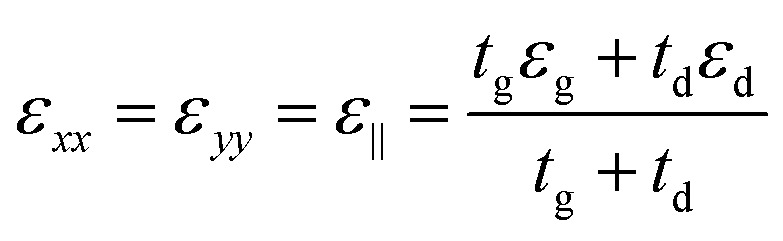
2
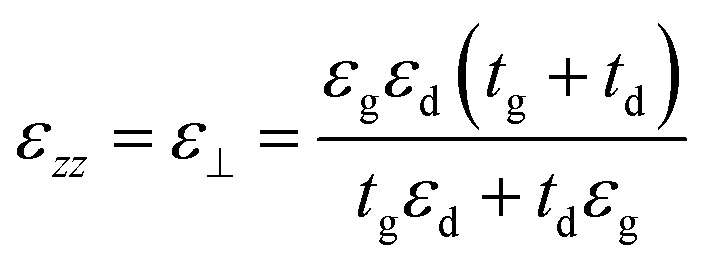
where ε_‖_ and *ε*_⊥_ are in-plane and out-of-plane permittivity components, respectively. The effective permittivity and thickness of graphene are represented by *ε*_g_ and *t*_g_ and that of the dielectric by *ε*_d_ and *t*_d_, respectively. Due to the atomically thin nature of graphene, *t*_g_ ≪ *t*_d_ and hence we can approximate, *ε*_⊥_ ≅ *ε*_d_. Since the period (=*t*_g_ + *t*_d_) of the multilayer graphene/Al_2_O_3_ HMM structure is much smaller than the operating wavelength, it can be considered as an anisotropic metamaterial.^44^ For Al_2_O_3_ and Au, the permittivity values are directly taken from the Lumerical material database while, *ε*_g_ can be expressed as:^45^3
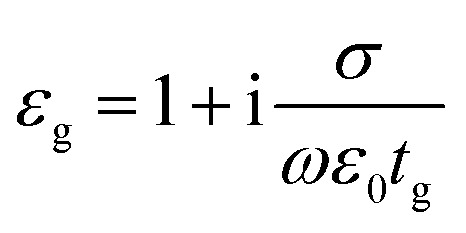
where, *ε*_0_ and *σ* are permittivity of vacuum and surface conductivity of graphene, respectively. This relation assumes that the electronic band structure of a graphene sheet is not affected by the neighbouring sheets. Without considering external magnetic field, the isotropic surface conductivity of graphene is calculated by using the Kubo formula where, *σ* is expressed as the summation of intraband (*σ*_intra_) and interband (*σ*_inter_) transitions as:^45^4
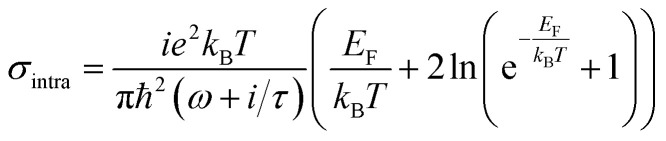
5
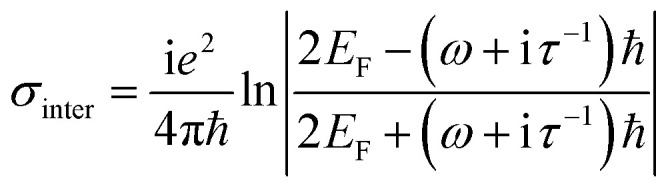
where, *ω* is the frequency of the incident light, *τ* is the electron–phonon relaxation time, *E*_F_ is the Fermi energy, *T* designates the temperature in Kelvin and *e*, *k*_B_ and *ℏ* represent electronic charge, Boltzmann constant and reduced Planck's constant, respectively. The relaxation time is given by, *τ* = *μE*_F_/*ev*_F_^2^, with *v*_F_ as the Fermi velocity and *μ* as the carrier mobility. The Fermi energy depends on the 2D carrier density (*η*_2D_) of graphene sheets, such that *E*_F_ = *ℏv*_F_ (π*η*_2D_)^1/2^ and can be tuned by applying an external voltage. Between, *λ* = 3 to 10 μm, *σ* is calculated by taking *T* = 300 K, *μ* = 10 000 cm^2^ (V s)^−1^ and *v*_F_ = 10^6^ m s^−1^. Fig. 1(d) plots the real and imaginary parts of the effective permittivity for the proposed device.^16,45^ The uniaxial dielectric tensor components, of the anisotropic graphene/Al_2_O_3_ HMM stack, are determined with EMT, where the graphene/dielectric bilayers satisfy the criterion of EMT. The background RI is set at 1.33 (water). The FDTD simulations are carried out with *N* = 11, *g* = 30 nm, *p* = 820 nm, *d* = 410 nm, and *E*_F_ = 0.64 eV. Hyperbolic dispersion is observed for wavelengths of *λ* > 3.58 μm, where *ε*_‖_ (*ε*_*xx*_ = *ε*_*yy*_) < 0 and *ε*_⊥_ (=*ε*_*zz*_) > 0. When *λ* < 3.58 μm, both *ε*_‖_ > 0 and *ε*_⊥_ > 0, and the dispersion is elliptical. Since the energy of the incident photon, *hω* ≪ 2*E*_F_, it forbids interband transitions in graphene and therefore, the contributions from *σ*_inter_ can be ignored.

Eqn (1)–(5), illustrate that the surface conductivity of graphene, in the graphene/Al_2_O_3_ HMM structure, changes with the Fermi energy and the permittivity or refractive index of the surrounding environment. The change in *σ* affects the optical properties of graphene and consequently, tunes the resonance wavelength of the HMM structure. The biomolecules, adsorbed onto the graphene/Al_2_O_3_ stack, are modelled with a wide range of RIs such as 1.41 (human g-immunoglobulin or IgG), 1.445 (human serum albumin or HSA), 1.462 (single-stranded DNA or ss-DNA) and 1.53 (double-stranded DNA or ds-DNA).^26^ The resonance wavelength shifts, caused by these commonly studied biomolecules, are obtained using the FDTD method. Aqueous medium (RI = 1.33) has been used as the reference.

## 3. Results and discussion

### 3.1 Optimization of the graphene/Al_2_O_3_ HMM structure

**Fig. 2:**
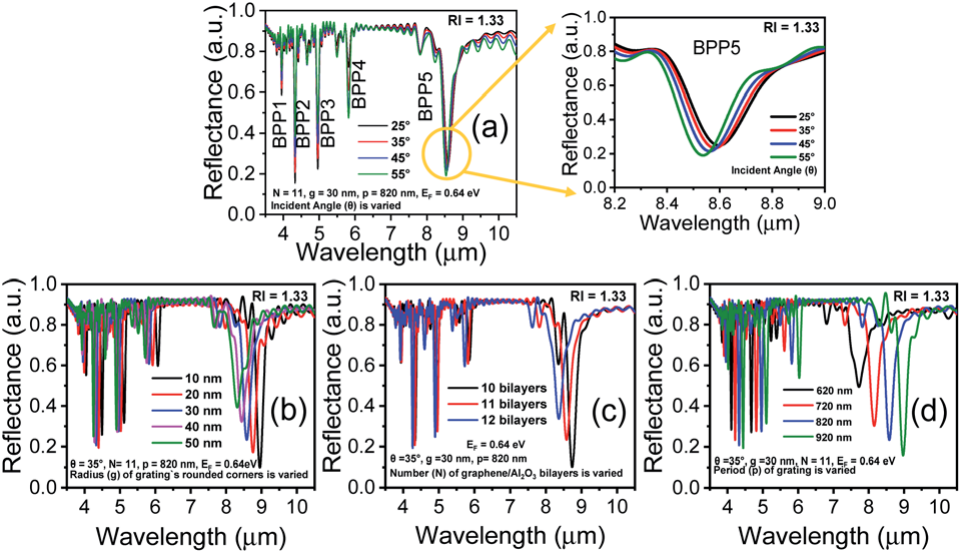
Reflectance spectra of the grating coupled graphene/Al_2_O_3_ HMM structure for varying (a) angles of incidence *θ*, with *g* = 30 nm, *N* = 11 and *p* = 820 nm. Enlarged view shows blue shift of the resonance wavelength, with increasing *θ* for BBP5 (b) radius (*g*) of the rounded corners in the grating, with *θ* = 35°, *N* = 11, and *p* = 820 nm (c) number of graphene/Al_2_O_3_ bilayers (*N*), with *θ* = 35°, *g* = 30 nm, and *p* = 820 nm (d) grating period (*p*) with *θ* = 35°, *g* = 30 nm, and *N* = 11. In all cases, *E*_F_ = 0.64 eV.

Fig. 2(a)–(d) show how the optical properties of graphene/Al_2_O_3_ HMM structure can be tuned by changing the angle (*θ*) of the TM polarized light, the radius (*g*) of the rounded grating corners, the number of graphene/dielectric bilayers (*N*) and the period (*p*) of the underlying grating structure, respectively. In the hyperbolic region (*λ* > 3.58 μm) shown in Fig. 2(a), five reflectance dips are observed, which represent the high-k modes. At, *θ* = 35°, the high-k modes correspond to wavelengths of 3.95 μm (BPP1), 4.33 μm (BPP2), 4.96 μm (BPP3), 5.82 μm (BPP4) and 8.58 μm (BPP5). Considering zero transmission through the grating coupled HMM, near about 85% absorption is obtained for BPP2, while BPP3 and BPP5 show ∼80% absorption. Modes corresponding to BPP1 and BPP4, on the other hand, absorb ∼40% and ∼50% of the incident TM polarized light, respectively. Furthermore, the fifth high-k mode (BPP5) shows broader and more distinct resonance dips than the rest of the modes. Strong absorption by HMMs is attributed to the indefinite hyperbolic *k*-space, which results in large number of photonic density of states inside the HMM. The reflection minima represent the highly confined BPPs, which are slightly blue shifted, with increasing angle of incidence. Higher incident angle of the propagating light tends to result in greater contribution of the signal component parallel to the interface. The blue shift is more obvious for longer wavelength modes, as shown in Fig. 2(a) for BPP5, and is caused by the change in effective index (modal index, *n*_modal_) of BPPs with incidence angle. For the grating coupled structure, *n*_modal_ is given by:^46^6
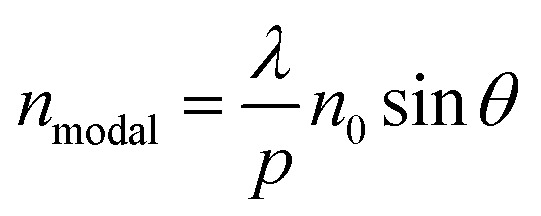
where *λ* is the incident wavelength, *p* is the grating period, *n*_0_ is the refractive index of the incident medium and *θ* is the incident angle. At higher angle of incidence, the resonant wavelength decreases (blue shift), for a given value of *n*_modal_. Fig. 2(b) and (c) depict the influence of the radius (*g*) of the rounded corners in the grating structure and the number (*N*) of graphene/Al_2_O_3_ bilayers, respectively, on the reflectance spectra. In both cases, absorption decreases, and the resonance shifts towards the shorter wavelengths as *g* and *N* increase. The shift in reflectance minima, with increasing *N*, is due to the coupling of the individual graphene plasmon modes. Increasing the grating period (*p*) causes a red shift of the resonance wavelengths and increases the depth of the reflectance dips, as displayed in Fig. 2(d). The associated electric field and absorption can, therefore, be enhanced by choosing appropriate values of *p* as shown by Chang *et al.*^42^ To obtain an optimized sensor performance, by detecting the resonance wavelength shift, the subsequent FDTD simulations are carried out with, *θ* = 35°, *N* = 11, *g* = 30 nm and *p* = 820 nm.

### 3.2 Tunable Fermi energy of graphene

**Fig. 3:**
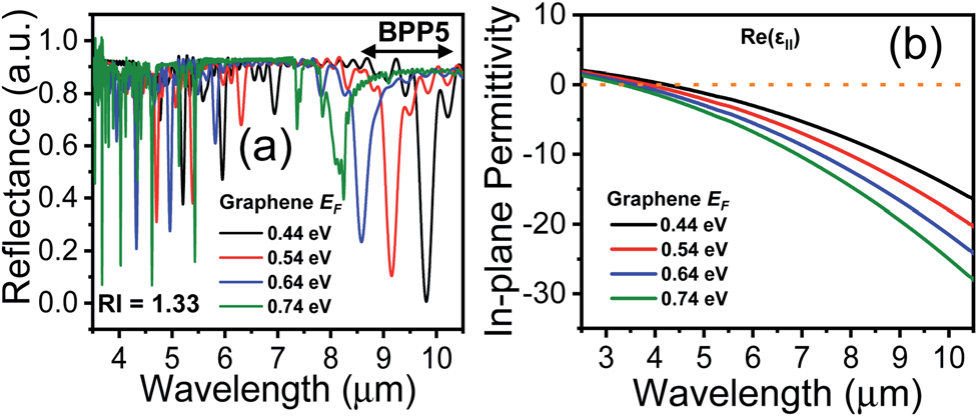
(a) Reflectance spectra of grating coupled graphene/Al_2_O_3_ HMM stack for different values of graphene Fermi energy *E*_F_, showing all five BPP modes (b) real part of in-plane permittivity, *ε*_‖_, with varying *E*_F_. Here, *θ* = 35°, *g* = 30 nm, *N* = 11 and *p* = 820 nm.

A feasible scheme for tuning the conductivity of graphene, *via* electrical biasing, has been demonstrated by Chang *et al.*,^36^ where the chemical potential of graphene was efficiently tuned by applying a bias voltage across each graphene layer. A similar scheme can be adopted, in our proposed HMM structure, to electrically bias the graphene layer, sandwiched between two layers of Al_2_O_3_. The voltage-controlled Fermi energy, *E*_F_, can be used to tailor the surface conductivity of graphene and hence control the absorption properties of the graphene/Al_2_O_3_ HMM structure.^47,48^ This is demonstrated in Fig. 3(a), where each *E*_F_ value corresponds to a specific doping level in graphene. The absorption decreases, and the resonant wavelengths undergo a blue shift, as *E*_F_ increases. Fig. 3(b) displays the real part of *ε*_‖_*vs.* incident wavelengths, plotted for different values of *E*_F_. The hyperbolic region is seen to shift towards shorter wavelengths (blue shift), as *E*_F_ increases, further confirming the strong dependence of the HMM's optical characteristics on the Fermi energy of graphene.

### 3.3 Evaluation of sensor performance

**Fig. 4:**
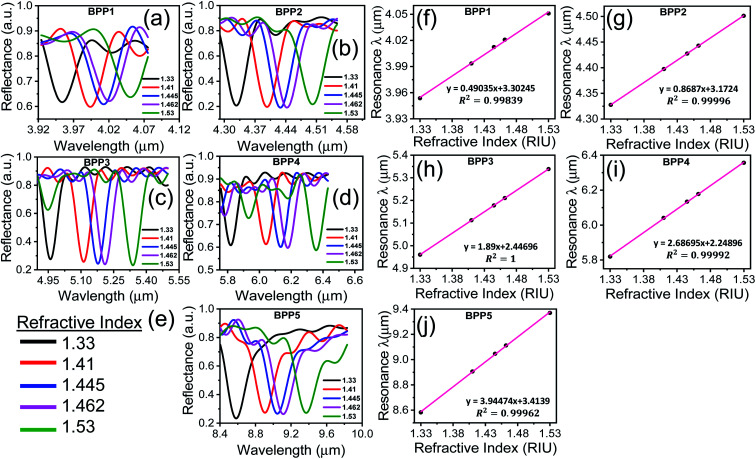
(a–e) Reflectance spectra of the graphene/Al_2_O_3_ HMM biosensor, detecting biomolecules with different RIs. (f–j) Corresponding linear fitting of resonant wavelength *versus* ambient RI. In all cases, *E*_F_ = 0.64 eV, *θ* = 35°, *g* = 30 nm, *N* = 11, and *p* = 820 nm.

The sensor performance is evaluated by calculating the resonance wavelength shift, for each high-k mode, in presence of the biomolecules represented by their respective RIs. Fig. 4(a)–(e) show the reflectance spectra for each BPP mode, for different RIs. An increase in RI induces a red shift in the resonance wavelength of each mode, as demonstrated earlier.^49^ The corresponding linear relationship, between the resonant wavelength and the RI of the surrounding medium, are displayed in Fig. 4(f)–(j). In each case, nearly perfect linear response is obtained which is indicative of excellent sensor quality. The sensitivity (*S*) of the graphene/Al_2_O_3_ HMM biosensor is given by:^16^7
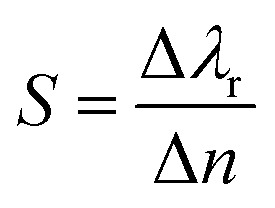
where, Δ*λ*_r_ is the resonance wavelength shift caused by Δ*n* change in refractive index of the surrounding medium, with respect to water (RI = 1.33) as the background. The resonance wavelength shift, relative to the reference solution, is calculated for commonly found biomolecules such as human g-immunoglobulin (IgG, RI = 1.41), human serum albumin (HSA, RI = 1.445), single-stranded DNA (ss-DNA, RI = 1.462) and double-stranded DNA (ds-DNA, RI = 1.53).

**Fig. 5:**
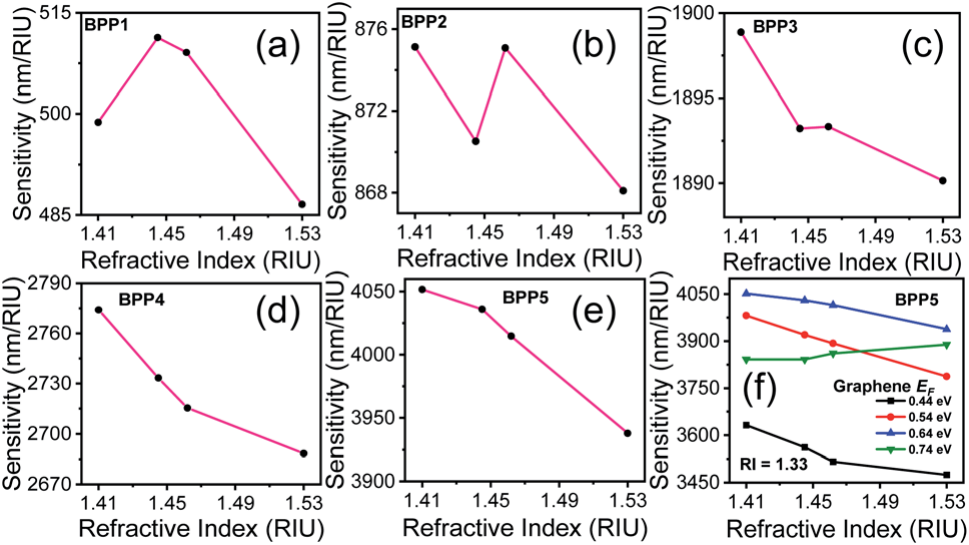
(a–e) Sensitivity of graphene/Al_2_O_3_ HMM biosensor for detecting biomolecules, represented by their respective RIs. The reference RI = 1.33 (water). In all cases, *E*_F_ = 0.64 eV, *θ* = 35°, *g* = 30 nm, *N* = 11, and *p* = 820 nm (f) sensitivity of graphene/Al_2_O_3_ HMM biosensor, for the BPP5 mode, at different *E*_F_ of graphene, when RI = 1.33.

The sensitivity of the proposed biosensor depends on the BPP modes used for detection. Fig. 5(a)–(e) present the sensitivities for each mode, at specific RIs, corresponding to the biomolecules. It is noted that, the sensitivity decreases with increase in RI as the resonance wavelengths undergo red shifts. BPP5 provides the highest detection sensitivity, with sensitivities ranging between 4052 nm RIU^−1^ and 3938 nm RIU^−1^ for IgG (RI = 1.41) and ds-DNA detection (RI = 1.53), respectively. The corresponding RI sensitivities for the other BPP modes are 499 nm RIU^−1^ (RI = 1.41) and 487 nm RIU^−1^ (RI = 1.53) for BPP1, 875 nm RIU^−1^ (RI = 1.41) and 868 nm RIU^−1^ (RI = 1.53) for BPP2, 1899 nm RIU^−1^ (RI = 1.41) and 1890 nm RIU^−1^ (RI = 1.53) for BPP3 and finally, 2774 nm RIU^−1^ (RI = 1.41) and 2688 nm RIU^−1^ (RI = 1.53) for BPP4.

The sensitivity can be tuned by varying the Fermi energy (*E*_F_) of graphene, through an external bias. This is demonstrated in Fig. 5(f), which shows the sensitivity for BPP5 mode, at different values of *E*_F_, in aqueous solution (RI = 1.33). The highest sensitivity is achieved for *E*_F_ = 0.64 eV. The other high-k modes also follow the same trend. Hence, we used *E*_F_ = 0.64 eV, for evaluating the performance of the proposed biosensor.

**Fig. 6:**
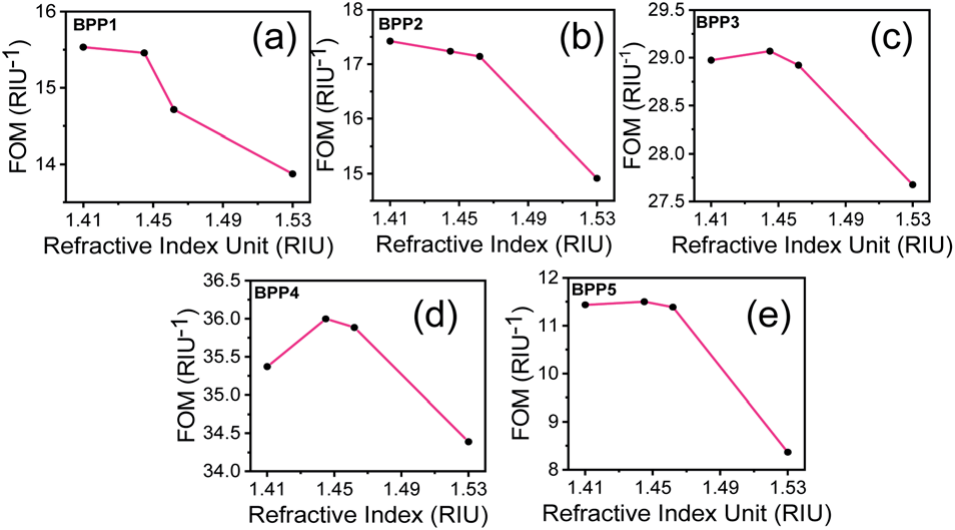
(a–e) FOM values of graphene/Al_2_O_3_ HMM biosensor for detecting biomolecules as represented by their respective RIs. In all cases, *E*_F_ = 0.64 eV, *θ* = 35°, *g* = 30 nm, *N* = 11, and *p* = 820 nm. The reference RI = 1.33 (water).

The sensor performance, in the detection limit, is determined by the sharpness of the resonance dips, as quantified by the FOM parameter:^16^8
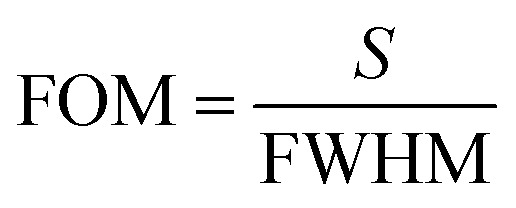
where, *S* is the sensitivity and FWHM is the full width of the resonance dip at half-maximum. Here, the detection accuracy is defined as the reciprocal of FWHM. A sensor with large FOM has high detection accuracy and produces resonance dips with narrow line width. This enables the sensor to detect small RI changes in the sensing medium with high sensitivity. The FOM values, for each of the BPP modes, are shown in Fig. 6(a)–(e) at specific RIs. In general, high sensitivities and sharp resonance dips, results in higher FOMs, for smaller RIs. Between RI = 1.41 (IgG) and 1.53 (ds-DNA), the FOM values are 15.03/RIU (RI = 1.41) and 13.37/RIU (RI = 1.53) for BPP1, 17.42/RIU (RI = 1.41) and 14.91/RIU (RI = 1.53) for BPP2, 28.98/RIU (RI = 1.41) and 27.68/RIU (RI = 1.53) for BPP3, 35.37/RIU (RI = 1.41) and 34.39/RIU (RI = 1.53) for BPP4 and 11.44/RIU (RI = 1.41) and 8.37/RIU (RI = 1.53) for BPP5. Based on the sensitivity and FOM values, a trade-off must be made between high detection sensitivity and high accuracy, when designing the biosensor. For example, for IgG detection (RI = 1.41), using BPP5 gives 1.5× higher RI sensitivity than BPP4 because it provides a larger spectral shift as compared to BPP4 for the same change in RI. However, BPP5 produces wider reflectance dips, with nearly 4.5× the FWHM of BPP4. Hence, FOM for BPP5 is three-folds lower than BPP4. A good balance between sensitivity (2774 nm RIU^−1^) and detection accuracy (FOM = 35.37/RIU) is, therefore, obtained for the BPP4 high-k mode.

**Fig. 7:**
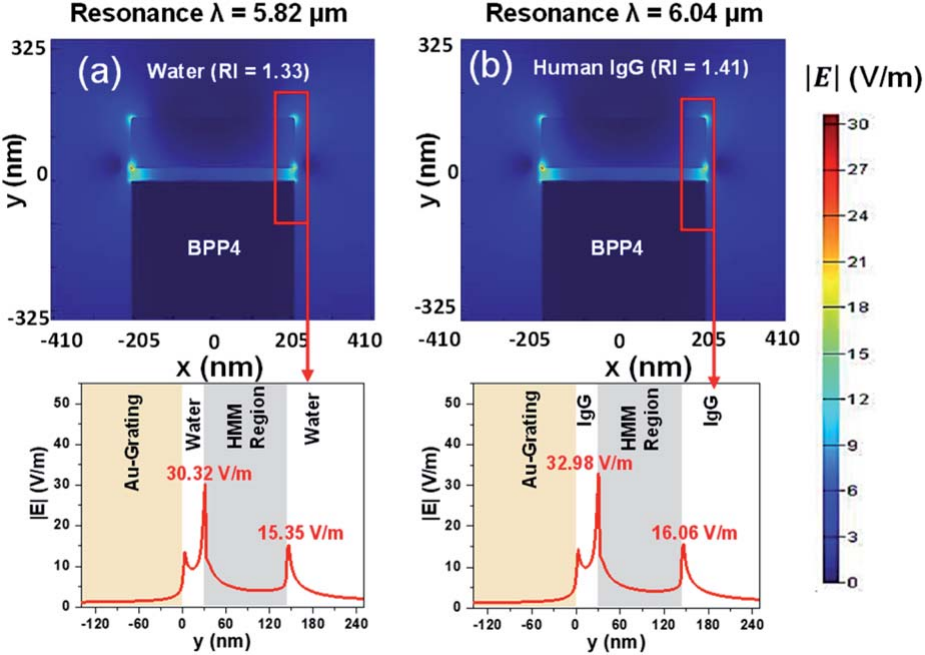
The distribution of electric field intensity, for BPP4, with (a) background RI = 1.33 and (b) background RI = 1.41. In all cases, *E*_F_ = 0.64 eV, *θ* = 35°, *g* = 30 nm, *N* = 11, and *p* = 820 nm.

For each BPP mode, the electric field intensity, surrounding the HMM structure, has been derived. To calculate the electric field intensity distribution, the HMM structure was transformed into a homogeneous medium, using the effective medium theory (EMT). This is made possible because the period of the HMM structure is in the sub-wavelength range.^42,50^Fig. 7(a) and (b), show the |*E*| field plots at the resonance wavelengths of 5.82 μm (RI = 1.33) and 6.04 μm (RI = 1.41) for BPP4. The BPP4 mode is chosen because it exhibits a good balance between sensitivity and FOM. From Fig. 7(a) and (b), it is observed that the |*E*| field is enhanced at the interface of the HMM structure and the surrounding medium, but decays rapidly within the HMM slab. Also, the interface |*E*| fields change with the change in background RI. The other BPP modes (not shown here) were found to generate similar electric field patterns around the HMM structure, with varying electric field strengths.

The performance of the proposed sensor has been benchmarked against some of the existing, state-of-the-art, graphene based plasmonic biosensors, as shown in Table 1. Several aspects of the sensors, including the sensing structure, RI detection range, maximum sensitivity and FOM have been compared. It can be concluded, from Table 1, that the proposed sensor performs better, in terms of maximum sensitivity. The FOM of the proposed sensor is 11.44/RIU at the highest sensitivity of 4052 nm RIU^−1^ for BPP5 mode. However, as described earlier, a FOM of 35.37/RIU can also be obtained, at a slightly reduced sensitivity of 2774 nm RIU^−1^, for BPP4 mode, when higher detection accuracy is desired. The highest FOM for BPP4 is attributed to the sharpest resonance peak, while, the highest sensitivity, for BPP5 results from the maximum shift in resonance wavelength for a unit change in RI. Although, metal/dielectric based HMM biosensors, as demonstrated by Sreekanth *et al.*^30^ can achieve sensitivities between 10 000 nm RIU^−1^ and 30 000 nm RIU^−1^, the detection is limited to visible and NIR wavelength regions. Graphene based HMM biosensors, on the other hand, offers mid-IR sensing capabilities in addition to external tunability, extreme scalability and lower loss.

**Table 1:** Comparison of sensing performance of graphene based plasmonic biosensors

Sensing structure	RI range	Maximum sensitivity (nm RIU^−1^)	FOM (RIU^−1^)	References
Hybrid metal–graphene plasmonic sensor	1–1.4	2300	28.75	16
LSPR based U-shaped multimode fiber with 3D complex of gold nanoparticles and multilayer graphene	1.340–1.352	1251.44	—	18
Standard single-mode fiber (SMF), laterally polished and coated with a thin gold film, with a single graphene sheet on top of the gold film	1.333–1.3505	1039.18	—	22
	1.3326–1.3497	413.79	—	
Graphene oxide/silver coated polymer cladding silica fiber	1.333–1.3731	3311	24	23
	1.3328–1.3739	2875	—	
Graphene film patterned into periodic arrays of nanoribbons on top of a transparent substrate	1.312–1.530	1697	∼3	26
Graphene/Al_2_O_3_ HMM on top of Au grating structure	1.330–1.530	4052	11.44	This work

## 4. Conclusions

In summary, we proposed a graphene/Al_2_O_3_ HMM stack, coupled to Au grating structure, for tunable, label-free, and highly sensitive biosensing in the mid-IR region. The absorption characteristics, of the HMM stack, can be tuned by changing the Fermi energy of graphene, using an external voltage, or by changing the structure of the HMM stack. The wavelength dependent effective permittivities, of the HMM layer, are obtained from the well-established EMT. The sensor performance has been numerically evaluated, through FDTD simulations, by determining the resonance wavelength shift, caused by the change in RI of the surrounding medium, where different RIs correspond to different biomolecular entities. The proposed sensor can detect commonly found biomolecules (IgG, HSA, ss-DNA, and ds-DNA) with detection sensitivities as high as 4052 nm RIU^−1^ and a corresponding FOM of 11.44/RIU. Both the sensitivity and FOM values of our proposed sensor are either higher or comparable to the previously reported graphene based plasmonic biosensors. HMM structures, like the one proposed here, can be fabricated and characterized using well-established techniques, as demonstrated by Chang *et al.*,^36,42,51^ paving the way for the next generation of high-performance, graphene based, mid-IR biosensing platforms.

## Author contributions

R. Ahmed and M. Hossain conceived the idea. S. Cynthia carried out all the simulations and calculations. Both M. Hossain and S. Cynthia interpreted the results and wrote the manuscript. R. Ahmed, S. Islam and K. Ali provided useful suggestions to improve the scientific content of the manuscript.

## Conflicts of interest

There are no conflicts to declare.

## Supplementary Material
